# The impact of stress-coping strategies on perceived stress during the COVID-19 pandemic among university students an interventional study

**DOI:** 10.1186/s12888-023-04730-y

**Published:** 2023-07-13

**Authors:** Asmaa Younis Elsary, Naglaa A. El-Sherbiny

**Affiliations:** Faculty of Medicine, Fayoum University, Fayoum, Egypt

**Keywords:** COVID-19, Student, Stress, Coping, Pandemic

## Abstract

**Background:**

Coronavirus (COVID-19) pandemic is a public health emergency. During the outbreak, a broad range of psychological disorders affected people at the individual, community, and international levels. This study aimed to assess the role of stress-coping strategies in relieving perceived stress among university students during the COVID-19 pandemic.

**Methods:**

This interventional study was nested on a cross-sectional design and involved students at Faiyum University in 2022.

**Results:**

Out of a sample of 2640 students, 2176 (82.4%) experienced moderate perceived stress, while 56 (2.1%) had more severe levels. Being female, nonmedical students, and rural inhabitants having a low socioeconomic status were associated with scores for severe and moderate levels of perceived stress. Among the interventional group, Modified Perceived Stress Scale scores significantly decreased after the implementation of the stress-coping program, with a p value < 0.001. Improvements in perceived stress levels were observed among male, medical, and high-socioeconomic-status students.

**Conclusion:**

Perceived stress levels were associated with being female, engaging in nonmedical study, and having low socioeconomic status and decreased after a stress-coping program was implemented. These findings assert the need to develop regular campaigns to provide psychological support and stress-coping strategies that may help students overcome different stressors.

## Introduction

The coronavirus disease (COVID-19) is a serious infectious respiratory illness. The World Health Organization (WHO) reported **619,161,228** confirmed cases of COVID-19, including **6,537,636** deaths, since the beginning of the pandemic in different countries with global vaccine doses of **12,723,216,322**. It was considered a public health emergency with the greatest challenges facing the global community [1].

COVID-19 infections influence the psychological profile of the general population, especially those who experience the illness themselves, fear a serious complication, or even panic from transmitting the disease to their families and those who are afraid of the COVID-19 social stigma even after full recovery [2]. The pandemic has caused a significant increase in psychiatric and behavioral disorders that could end with the patient considering suicide [3]. Governments had to implement some quarantine measures such as pausing the academic year and closing businesses leading to the collapse of income and even loss of jobs, with no information about the return to normal life [4].

The pandemic has also placed an unprecedented amount of strain on students, universities, and the educational system. Colleges faced the difficult choice of offering classes online to avoid diminishing their ability to serve their students [5]. Overall, the findings and respondents’ open-ended remarks about professors’ personal experiences and concerns about the future demonstrate the “degree of worry and scrambling” that many teachers experienced throughout the shift to online education [6].

Stress response can depend on an individual’s background, family, social support from friends, financial situation, health and emotional background, the community in which they live, and many other factors. Anyone can be affected by the potential changes due to the COVID-19 pandemic and the actions taken to stop the spread of the virus [7].

Many studies have evaluated stress and anxiety during the COVID-19 pandemic, the majority of which concentrating on healthcare workers and patients. With respect to the youth or university students, research has been scant. Universities had to shift their teaching and assessment processes to online methods, which disrupted all academic activities and put students under many stressors; in fact, some incidents of suicide among university students occurred during and after the COVID-19 period because of stress.

For these reasons, the current study investigated levels of perceived stress among university students and tested the effect of a stress-coping program in relieving their stress. It could help control stress caused by any stressors that students are exposed to.

## Methods

### Design and setting of the study

**Fig. 1:**
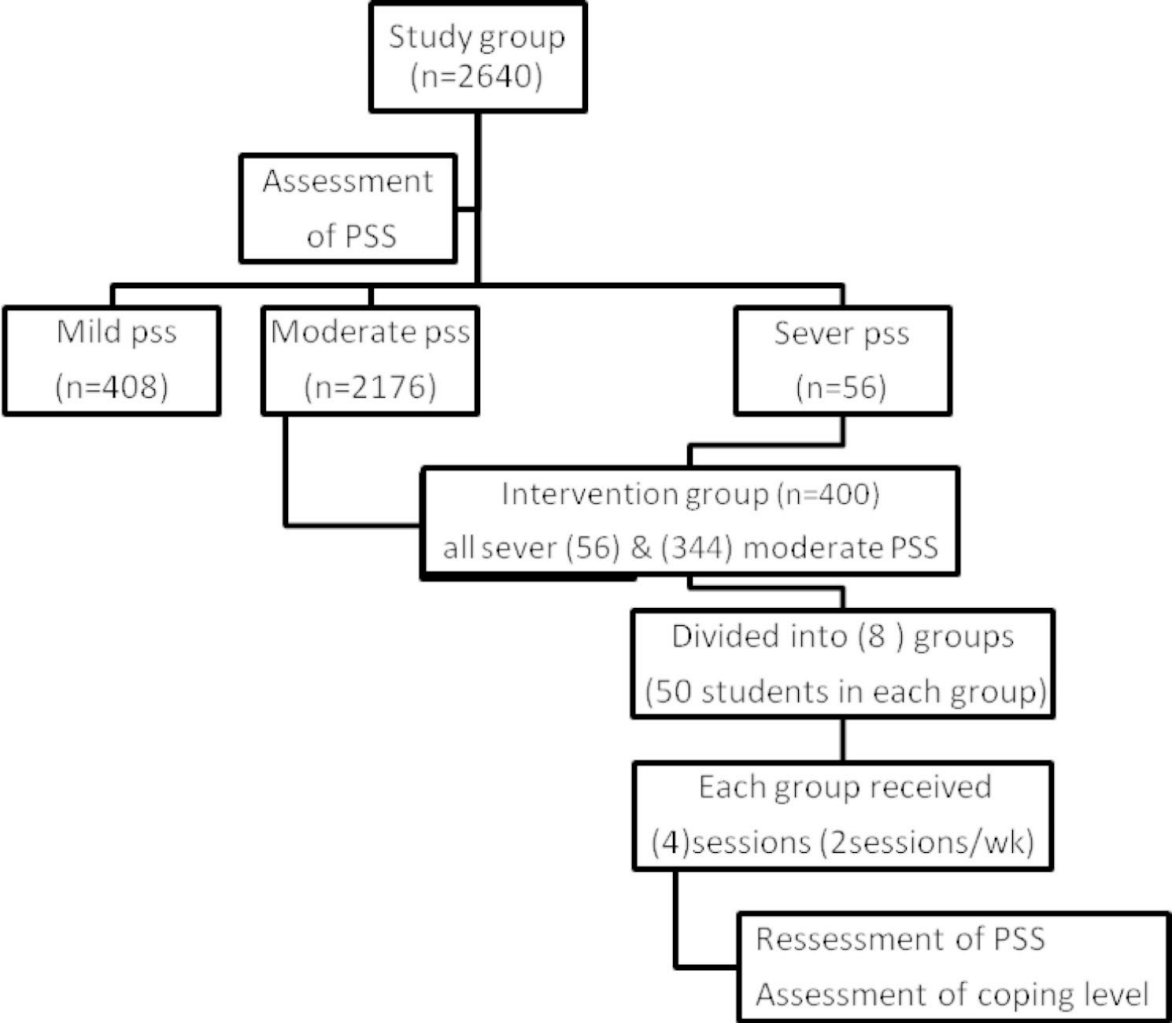
Flow chart of the study framework

This interventional study, conducted at Faiyum University, Egypt, was nested on a cross-sectional design. It has two stages: a cross-sectional stage involving **2640** Faiyum University students to assess the prevalence of perceived stress levels, and an interventional stage that enrolled 400 students who had moderate or severe levels of stress at the first stage. They then underwent a stress-coping program to test its impact on their perceived stress and coping levels. (Fig. 1)

### Sampling and participants

The sample size was calculated according to Epi Info 2000, at a confidence interval of 95% and a precision of 2%. The sample was increased by 10% to overcome problems associated with no responses and missing data. The study had a power of 85%. Its sample type was a multistage cluster random sample. At the first stage, six medical and nonmedical faculties were selected. At the second stage, two grades in each faculty were selected randomly. The final stage included two selected section/tutorial groups from each grade.

### Study tool and material

The students answered a self-administered Arabic questionnaire divided into three parts. The first part had five items on sociodemographic characteristics such as age, sex, residence, type of faculty, and grading. The second part assessed the students’ socioeconomic level using the **socioeconomic status scale**. The scale included 10 questions (parents’ educational background and occupation, family domain, home sanitation, and economic domain) with a total score of 48. Socioeconomic status was classified as low if the participants achieved less than 40% of the total score with a range of 0–19.2, medium if they achieved 40–70% of the total score ranging between 19.2 and 33.6, and high if they attained higher than 70% of the total score (between 33.6 and 48) [8].

The third part featured the **modified perceived stress scale (PSS)** as a psychological tool. The current study used 10 questions with 6 points reflecting the negative effect and 4 points estimating the positive effect of coping with stress. Scoring followed a 5-point scale: “never” (0), “seldom” (1), “sometimes” (2), “often” (3), and “very often” (4). Scores ranged from 0 (low perceived stress) to 40 (high perceived stress). The students were classified according to their total PSS scores: 0–13 (low perceived stress), 14–26 (moderate perceived stress), and 27–40 (high perceived stress) [9].

The questionnaire was developed in English and translated into Arabic with the help of a bilingual specialist. Another specialist revised the scale, and then a pilot study was conducted on 50 students to test the clarity of questions and assess the questionnaire’s validity and reliability.

### The intervention

The second stage of the study included 400 students with moderate and severe levels of perceived stress divided into 8 groups of 50 students each. Each group underwent two sessions of the stress-coping program per week for four sessions. The first session held an orientation about stressors (definition, types, and impact on mental and physical health). Afterward, an active stress-coping program was implemented for students. The researchers defined stress-coping strategies as methods utilized in circumstances where students experience a change in their environment or a stressor they cannot control. The coping program was based on four key strategies: **The first** strategy mainly focuses on identifying and managing all factors and sources of stress (problem-focused coping strategies) through action-oriented coping, aiming to change the source of stress. **The second** involves controlling stressor-related emotional responses using emotion-focused coping methods directed toward managing emotions. In this session, we taught individuals how to express their negative feelings, such as fear, anxiety, and depression. Each individual chose to either talk to an expert or write to express his or her emotions. **The third** focuses on avoidance behavior through which the participants ignore the problem and engage in other positive activities for mental disengagement. This is defined by a person’s conscious or unconscious efforts to avoid dealing with a stressor to protect themselves from the difficulties that it brings. **The fourth** key strategy is seeking and asking for help (e.g., social and family support, strengthening one’s religious faith by adopting a positive interpretation of events, etc.). Each session lasted between 30 and 45 min [10, 11].

The questionnaire was distributed to the intervention groups to estimate students’ perceived stress before and at the end of the stress-coping program. In addition, the **brief resilience scale (BRS)** was used to assess their coping level after finishing the program. The BRS included 6 items using a Likert scale from 1 to 5 with total scores ranging between 6 and 30. Total scores are divided by 6 to calculate the final score. Scores between 1 and 2.99 are interpreted as having low resilience, between 3 and 4.3 as having normal resilience, and between 4.31 and 5 as having high resilience [12, 13].

### Statistical analysis

Data analysis was conducted using the statistical package for the social sciences version 22 [14]. Independent-samples t-test was performed to compare quantitative measures between two independent groups. To compare the data of the three groups, a one-way analysis of variance test was used. In addition, a paired t-test was performed to compare two dependent quantitative data. For qualitative data, chi-square and McNemar’s test were used. P < 0.05 was considered statistically significant.

## Results

Among the 2640 students enrolled in the study, 488 (18.5%) were male while 2152 (81.5%) were female, 1648 (62.4%) lived in rural areas whereas 992 (37.6%) lived in urban areas, and 2184 (82.7%) were nonmedical students while 456 (17.3%) were medical students. Regarding socioeconomic status, 208 (7.9%) were classified as low, 1640 (62.1%) medium, and 792 (30%) high.

**Fig. 2:**
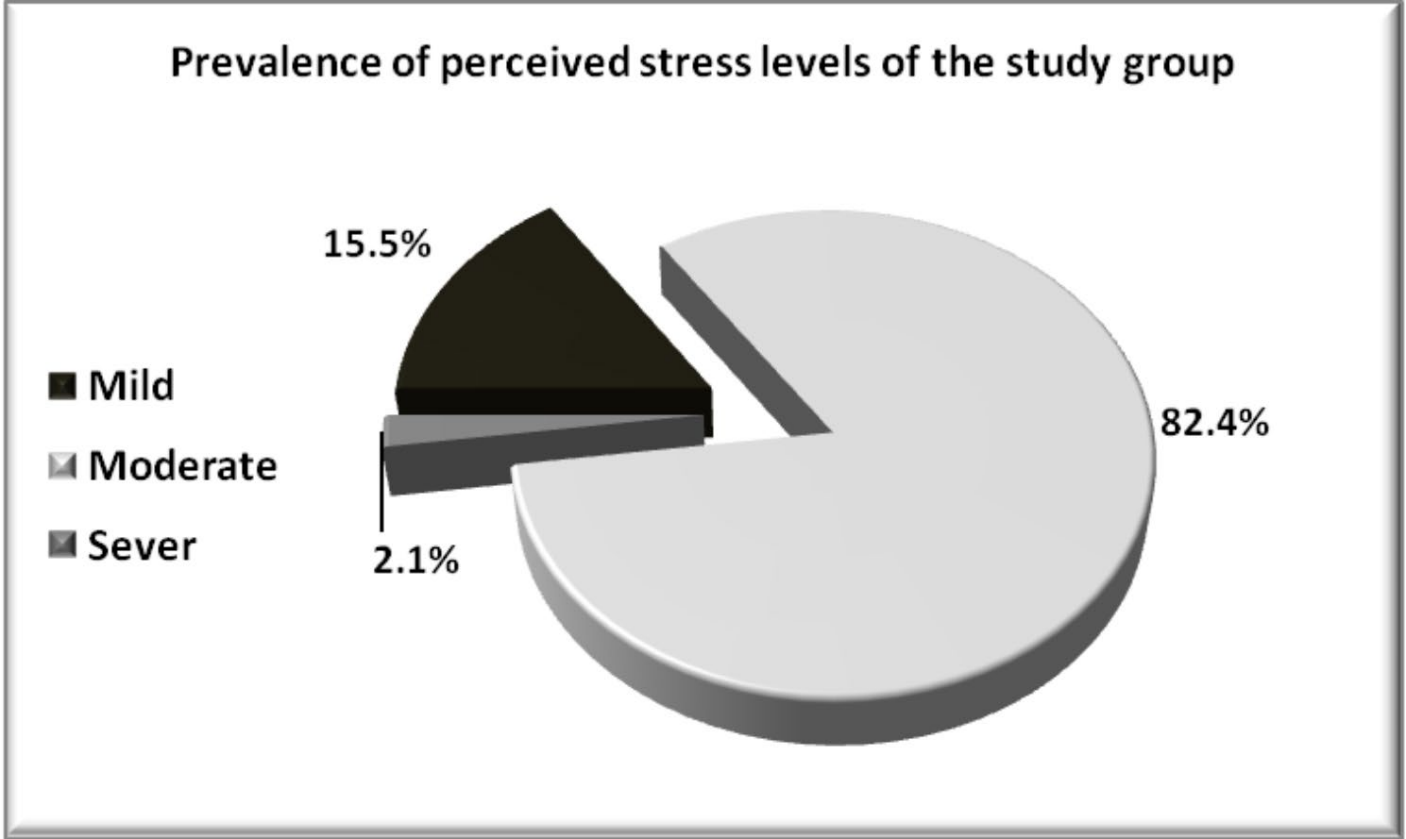
Prevalence of students’ Perceived stress levels of study group

The mean perceived stress score of the study group was 17.4 ± 4.2. The survey showed that 408 participants (15.5%) had a mild level of perceived stress, 2176 (82.4%) had moderate perceived stress, and 56 (2.1%) had severe perceived stress. (Fig. 2)

Statistically significant higher percentages of moderate and severe stress levels were observed among females, nonmedical students, rural inhabitants, and low-socioeconomic-status students (p < 0.001) (Table 1).

**Table 1:** Comparisons of PSS scores in different demographic characteristics among university students

Variables	PSS score			p value
	Low	Moderate	Severe	
	No (%)	No (%)	No (%)	
**Sex**				
Male	136 (27.9%)	344 (70.5%)	8 (1.6%)	< 0.001
Female	272 (12.6%)	1832 (85.1%)	48 (2.2%)	
**Type of study**				
Medical	112 (24.6%)	344 (75.4%)	0 (0%)	< 0.001
Nonmedical	296 (13.6%)	1832 (83.9%)	56 (2.6%)	
**Residence**				
Urban	183 (18.4%)	809 (81.6%)	(0%)	< 0.001
Rural	225 (13.7%)	1367 (82.9%)	56 (3.4%)	
**Socioeconomic level**				
Low	16 (7.7%)	184 (88.5%)	8 (3.8%)	< 0.001
Middle	216 (13.2%)	138 (84.4%)	40 (2.4%)	
High	176 (22.2%)	608 (76.8%)	8 (1%)	

### Data analysis for the intervention stage

Out of 400 students involved in the stress-coping program, 74 (18.5%) were male, and 326 (81.5%) were female. As for their type of study, 250 (62.5%) were medical students while 150 (37.5%) were nonmedical students. Regarding socioeconomic status, 248 (62%) were at the medium level, 120 (30%) at the high level, and only 32 (8%) at the low level.

Significant higher mean perceived stress scores were observed among females, nonmedical students, and low-socioeconomic-status individuals (p < 0.001) (Table 2).

**Table 2:** Comparisons of the interventional group’s PSS stress scores for different sociodemographic characteristics before undergoing the stress-coping program

Variables	PSS score before undergoing the stress-coping program	p value
	Mean ± SD	
Sex		
Male	20.3 ± 3.2	< 0.001
Female	28.2 ± 4.5	
**Type of study**		
Medical	21.3 ± 3.8	< 0.001
Nonmedical	29.2 ± 4.4	
**Socioeconomic level**		
Low	28.1 ± 4.1	0.001
Middle	24.3 ± 4.2	
High	19.8 ± 4.4	

After undergoing the program, the interventional group’s mean perceived stress scores decreased from 29.02 ± 4.3 to 18.4 ± 3.2. In addition, PSS scores significantly declined for each sociodemographic characteristic (sex, study type, and socioeconomic status) (Table 3).

Meanwhile, male, medical, and high-socioeconomic-status students showed much improvement in perceived stress levels and were more responsive to the stress-coping program.

**Table 3:** Comparison of the interventional group’s PSS stress scores for each sociodemographic characteristic before and after undergoing the stress-coping program

Variables	PSS score		p value
	Before	After	
	Mean ± SD	Mean ± SD	
**PSS score**			
Among study group	29.02 ± 4.3	18.4 ± 3.2	< 0.001
**Sex**			
Male	20.3 ± 3.2	15.5 ± 3.9	< 0.001
Female	28.2 ± 4.5	18.6 ± 4.2	< 0.001
**Type of study**			
Medical	21.3 ± 3.8	16.8 ± 4.4	< 0.001
Nonmedical	29.2 ± 4.4	19.5 ± 4.2	< 0.001
**Socioeconomic level**			
Low	28.1 ± 4.1	23.2 ± 3.9	0.001
Middle	24.3 ± 4.2	20.4 ± 4.2	< 0.001
High	19.8 ± 4.4	12.8 ± 4.2	< 0.001

After the participants’ engagement in the stress-coping program, we found a statistically significant increase in the percentage of those with low-level perceived stress and a decrease in the percentage of those with moderate and severe levels of perceived stress (p = 0.005) (Table 4).

**Table 4:** Comparison of the interventional group’s PSS stress levels before and after undergoing the stress-coping program

PSS score level	Follow-up				p value
	Before		After		
	No.	%	No.	%	
Low	----	-----	82	20.5%	**0.005**
Moderate	344	86%	298	74.5%	
Severe	56	14%	20	5%	

**Fig. 3:**
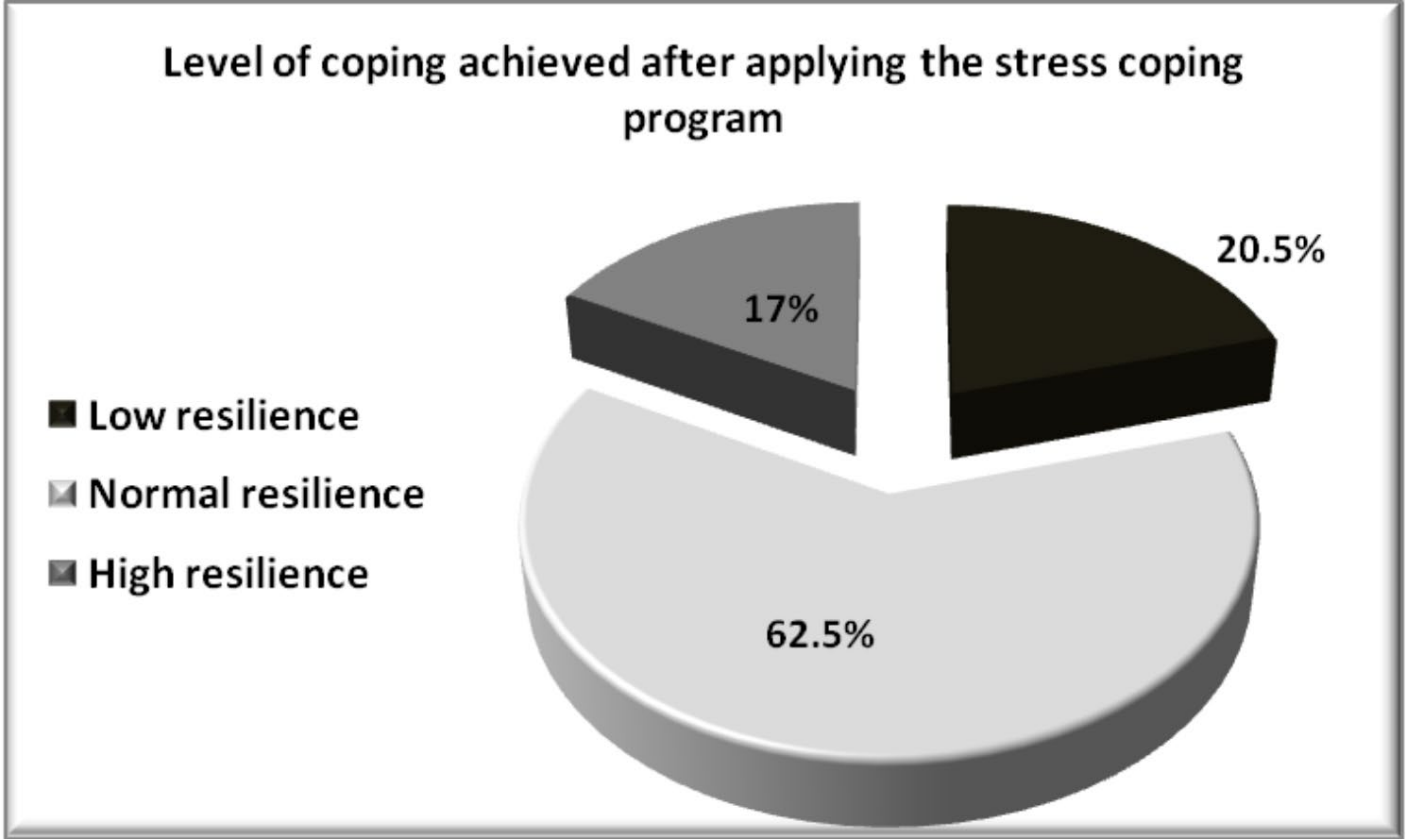
Students’ coping level achieved after undergoing the stress-coping program

After the implementation of the stress-coping program, 250 of the students (62.5%) achieved normal resilience levels, 68 (17%) achieved high resilience levels, but 82 (20.5%) achieved low resilience levels. (Fig. 3)

## Discussion

COVID-19 caused panic all over the world [15]. Feelings of stress, fear, confusion, or even anger during a crisis are normal phenomena. These are emotional responses to expected threats. The first step to managing stress and anxiety is to recognize it when it happens and not ignore it [16]. Infectious outbreaks and pandemics propagate fear and stress, which remain long after they are over, because the larger their scale, the greater the magnitude and impact of their psychological consequences [17].

The present findings are consistent with those of recent research showing moderate perceived stress levels among students [18, 19].

Among female participants, this study found statistically significant higher means of stress levels before and after they engaged in the stress-coping program. This could be explained by the higher incidence of stress-related disorders among females, such as anxiety and depression. In addition, females displayed different coping behaviors in response to stress [20, 21]. A higher incidence of stress and anxiety disorders among females owing to the increase in their stress response [22]. Also, a study conducted in China reported that during the COVID-19 outbreak and pandemic, stress, anxiety, and depression scores were higher among females and students [2].

A study in India found that stress is associated with being female and medical students because of academic, lifestyle, environmental, and social factors [23]. In accordance with previous findings, fear and stress levels increase during outbreaks and pandemics [24]. On the other hand, a study conducted in Egypt concluded that perceived stress had no predictive power with respect to gender or income. These results were in contrast with the current findings, which revealed a statistically significant higher mean of stress among females and people with low socioeconomic status [25].

The current study also showed a statistically significant association between perceived stress levels and low socioeconomic status, which was consistent with a study conducted in Denmark that reported a significant link between perceived stress and low income, low socioeconomic status, and unhealthy habits [26].

Low-income people are at a higher risk of psychological stress as well as respiratory diseases and infection because of poor housing and sanitary conditions, the absence of hand washing facilities, increased household crowding, and poor nutrition. In addition, people with low income usually cannot stay home and are likely late in seeking medical advice because of financial constraints [27]. Also, individuals who grew up in lower-socioeconomic-status families would be regularly exposed to external stimuli that might result in higher perceived levels of stress [28]. Preliminary findings from Belgium, the Netherlands, Switzerland, and the United Kingdom suggest that inequality has widened with the decline in learning. During the school closures, children from high socioeconomically levels received more parental support with their studies [29].

This study revealed a statistically significant link between perceived stress levels and nonmedical study, which is consistent with a study in China that revealed that being knowledgeable about the COVID-19 pandemic, may be a protective agent against perceived stress among medical staff [30].

The present study also showed a statistically significant decrease in perceived stress levels after implementing the stress-coping program. This improvement in perceived stress levels was in agreement with another Chinese study that reported that proper communication and training could reduce psychological stress during outbreaks and pandemics [23]. Another American study concluded that active coping strategies offer vital mental and physical protection against the negative effects of stress [28].

In addition, this study observed a statistically significant increase in cases of mild perceived stress compared with the decrease in cases of moderate and high-perceived stress after enrolling the study group in the stress-coping program. These findings were consistent with those of another Egyptian study that concluded that 70% of the study group showed moderate stress, 22% low stress, and 7% severe stress [23]. Another study in India found that the prevalence of stress among the study group was 24.42%; 10% had low stress, 7.6% had moderate stress, and 6.8% had severe stress [22].

The WHO reported that credible and trustworthy sources of information would help determine the risk so that reasonable precautions could be taken [31]. The Center for Disease Control and prevention (CDC) shared information and facts about COVID-19 with the population so that they could understand the risk and therefore feel less stress during an outbreak. However, information overload could aggravate stress and anxiety in the community [32] Furthermore, a Chinese study found that disclosing additional information about COVID-19 was significantly associated with lower stress, anxiety, and depression scores [2].

The current results are consistent with research showing the ability of stress-coping strategies to improve students’ resilience [33, 34].

This study’s strengths included its implementation of a stress-coping program and examination of its positive effect on improving perceived stress levels. It had some limitations, however, such as the difficulty in obtaining permits and the logistics of delivering all program sessions to reach an actual measure of stress during the COVID-19 outbreak. The participants received too much information in such a short time; one program was not enough to enable them to control their stress.

## Conclusion and recommendations

This study found moderate perceived stress levels among students. PSS was associated with being female, studying in nonmedical faculties, residing in rural areas, and having low socioeconomic status. Our results showed that perceived stress levels decreased and improved after the implementation of a stress-coping program. These findings highlight the need to develop regular campaigns to provide students psychological support and stress-coping strategies that would help them overcome stressors, especially during pandemics.

## Data Availability

The datasets used and/or analyzed during the current study are available from the corresponding author on reasonable request.
